# Machine Learning Reveals Impacts of Smoking on Gene Profiles of Different Cell Types in Lung

**DOI:** 10.3390/life14040502

**Published:** 2024-04-13

**Authors:** Qinglan Ma, Yulong Shen, Wei Guo, Kaiyan Feng, Tao Huang, Yudong Cai

**Affiliations:** 1 School of Life Sciences, Shanghai University, Shanghai 200444, China; mql1117@shu.edu.cn; 2 Department of Radiotherapy, Strategic Support Force Medical Center, Beijing 100101, China; shenyulong1087@163.com; 3 Key Laboratory of Stem Cell Biology, Shanghai Jiao Tong University School of Medicine (SJTUSM) & Shanghai Institutes for Biological Sciences (SIBS), Chinese Academy of Sciences (CAS), Shanghai 200030, China; gw_1992@sjtu.edu.cn; 4 Department of Computer Science, Guangdong AIB Polytechnic College, Guangzhou 510507, China; kyfeng@gdaib.edu.cn; 5 Bio-Med Big Data Center, CAS Key Laboratory of Computational Biology, Shanghai Institute of Nutrition and Health, University of Chinese Academy of Sciences, Chinese Academy of Sciences, Shanghai 200031, China; 6 CAS Key Laboratory of Tissue Microenvironment and Tumor, Shanghai Institute of Nutrition and Health, University of Chinese Academy of Sciences, Chinese Academy of Sciences, Shanghai 200031, China

**Keywords:** smoking, lung cell, gene expression profile, machine learning, marker, explainable artificial intelligence

## Abstract

Smoking significantly elevates the risk of lung diseases such as chronic obstructive pulmonary disease (COPD) and lung cancer. This risk is attributed to the harmful chemicals in tobacco smoke that damage lung tissue and impair lung function. Current research on the impact of smoking on gene expression in specific lung cells is limited. This study addresses this gap by analyzing gene expression profiles at the single-cell level from 43,539 lung endothelial cells, 234,349 lung epithelial cells, 189,843 lung immune cells, and 16,031 lung stromal cells using advanced machine learning techniques. The data, categorized by different lung cell types, were classified into three smoking states: active smoker, former smoker, and never smoker. Each cell sample encompassed 28,024 feature genes. Employing an incremental feature selection method within a computational framework, several specific genes have been identified as potential markers of smoking status in different lung cell types. These include *B2M*, *EEF1A1*, and *TPT1* in lung endothelial cells; *FTL* and *MT-ATP8* in lung epithelial cells; *HLA-B* and *HLA-C* in lung immune cells; and *HSP90B1* and *LCN2* in lung stroma cells. Additionally, this study developed quantitative rules for representing the gene expression patterns related to smoking. This research highlights the potential of machine learning in oncology, enhancing our molecular understanding of smoking’s harm and laying the groundwork for future mechanism-based studies.

## 1. Introduction

The well-documented and multifaceted deleterious impacts of tobacco smoking on pulmonary health encompass conditions ranging from impaired lung function to carcinogenesis [1,2,3]. Chronic exposure to tobacco smoke toxicants incites inflammatory and oxidative stress responses, precipitating obstructive and restrictive lung diseases, notably chronic obstructive pulmonary disease (COPD) [4,5,6]. Additionally, the carcinogenic compounds in smoke contribute to the increased incidence of both small-cell and non-small-cell lung cancer among smokers, owing to their role in inducing DNA damage, genetic mutations, and chromosomal instabilities [7,8,9]. Recent studies using machine learning methods to analyze smoking-associated transcriptome aberrations in blood have highlighted the broader genetic implications of smoking for lung health, indicating a complex interplay between genetic factors and environmental exposure [10]. Environmental factors, particularly air pollution, significantly impact lung health. Studies linking PM2.5 air pollution to lung cancer have shown that air pollutants can exacerbate the carcinogenic effects of tobacco smoke through changes in protein interactions and DNA methylation [11]. Even healthy lung cells are susceptible to these adverse effects, undergoing morphological and functional alterations that increase the risk of pathological transformations and disease onset [12,13]. While a significant body of research has addressed the lung carcinogenicity associated with smoking, further investigations are required to elucidate the molecular regulation impacts of smoking on the diverse cells constituting the human lung.

Tobacco smoking exerts significant adverse effects on various lung cells—including epithelial, endothelial, immune, and stromal cells—compromising pulmonary function and enhancing vulnerability to diseases. Epithelial cells exposed to smoke suffer from cellular stress, DNA damage, impaired mucociliary clearance, and heightened apoptosis [14,15,16]. Endothelial cells undergo increased oxidative stress and inflammation, resulting in augmented permeability and systemic inflammatory responses [17,18]. Smoking modifies the function of immune cells, especially macrophages and T-cells, leading to chronic inflammation and heightened susceptibility to infections and immune-related pulmonary ailments [14,19]. The extracellular matrix modifications in stromal cells induce tissue remodeling and fibrosis [20,21]. These cellular changes collectively contribute to the development of conditions like COPD, lung cancer, respiratory infections, and asthma among smokers.

The amalgamation of machine learning and single-cell data technologies has markedly advanced lung research, enabling nuanced analysis of cellular heterogeneity, gene expression patterns, and cellular interactions within lung tissues. Machine learning expedites the analysis of expansive datasets derived from methods like single-cell RNA sequencing. It plays a crucial role in pinpointing distinct cell types, states, and potential biomarkers and forecasts disease trajectories and responses to treatments with enhanced accuracy. Pioneering studies by Huang et al., Dohmen et al., and Yang et al. leveraged this combined approach to unveil biomarkers associated with lung cancer and COPD [22,23,24]. Furthermore, machine learning has been instrumental in smoking-related research, particularly in deciphering complex genomic patterns [25,26]. This interdisciplinary strategy is poised to make significant contributions to personalized medicine, unveiling intricate disease mechanisms and identifying novel therapeutic targets for a spectrum of lung disorders.

In this investigation, we utilized the Human Lung Cell Atlas (HLCA) core data from CELLxGENE, as provided by Sikkema et al. [27]. The dataset encompasses single-cell data procured from a cohort of healthy lungs, inclusive of tissue data from 107 individuals. We separated the smoking status (active, former, never) for analysis, drawing from epithelial, endothelial, immune, and stromal cell expression profiles. Each sample is characterized by 28,024 gene features. We deployed eight feature ranking algorithms—including adaptive boosting (Adaboost) [28], categorical boosting (CatBoost) [29], extremely randomized trees (ExtraTrees) [30], least absolute shrinkage and selection operator (LASSO) [31], light gradient boosting machine (LightGBM) [32], Monte Carlo feature selection (MCFS) [33], random forest (RF) [34], and extreme gradient boosting (XGBoost) [35]—to prioritize gene features associated with smoking. An incremental feature selection (IFS) approach [36] was adopted to pinpoint the features of paramount significance for classification with the help of two classification algorithms, decision tree (DT) [37] and RF [34]. In the subsequent phase, DTs were further adopted to derive rules for identifying smoking status. Despite progress, gaps remain in our understanding of how smoking impacts the transcriptional regulation in the microenvironment of lung tissues, particularly at the single-cell level. Our study advances knowledge by analyzing smoking-associated cellular changes, identifying novel biomarkers, and elucidating the interplay between smoking and lung cell functional alterations. These contributions aim to enhance the prevention and treatment of smoking-related lung diseases.

## 2. Materials and Methods

### 2.1. Gene Expression Data in Lung Cells Related to Smoking Status 

In this study, gene expression profile data were sourced from the CELLxGENE database as presented by Sikkema et al. [27]. Such data utilized single-cell RNA sequencing (scRNA-seq) and gathered comprehensive metadata across 14 datasets to create the HLCA. For data preprocessing, the HLCA integrated these scRNA-seq data and metadata, established a hierarchical framework for consistent cell type naming, and implemented multiple quality control measures. These measures included filtering based on gene and cell counts, mitochondrial gene and intronic reads percentages, log normalization, variable gene identification, principal component analysis, and clustering to refine the data and remove low-quality clusters [27]. The cells included in the specific four cell types are listed in Table S1. 

The utilized dataset comprised 43,539 lung endothelial cells, 234,349 lung epithelial cells, 189,843 lung immune cells, and 16,031 lung stromal cells. Each cell sample encompassed 28,024 feature genes. Within the endothelial cell subset, 6521 were from active smokers, 7189 from former smokers, and 29,829 from never smokers. The epithelial cell subset contained 37,905 from active smokers, 47,945 from former smokers, and 148,499 from never smokers. The immune cell set comprised 41,801 from active smokers, 34,853 from former smokers, and 113,189 from never smokers. Lastly, the stromal cell dataset included 3415 from active smokers, 2342 from former smokers, and 10,274 from never smokers. 

### 2.2. Feature Ranking Methods for Prioritizing Features Based on Their Importance

Our study delved into gene expression profiles of specific samples, discovering numerous genes, but only a few linked to smoking status. To gain a deeper insight into these smoking-associated genes, we employed eight feature ranking algorithms. These included Adaboost [28], CatBoost [29], ExtraTrees [30], LASSO [31], LightGBM [32], MCFS [33], RF [34], and XGBoost [35]. Each method provided a unique approach to ranking features, enhancing our understanding of the data and ensuring a thorough analysis of the significance of the identified genes.

#### 2.2.1. Adaptive Boosting

AdaBoost, which stands for “Adaptive Boosting”, is one of the first and most popular boosting algorithms used in the field of machine learning. It was introduced by Yoav Freund and Robert Schapire in 1995 [28]. AdaBoost focuses on improving the predictions of the samples where previous classifiers (referred to as “weak learners”) made errors. AdaBoost, like other ensemble algorithms, can provide insights into feature importance, helping model interpretation and feature selection. The way AdaBoost determines feature importance is intrinsically linked to its base learner. Given that AdaBoost often employs DTs (usually shallow ones, like stumps) as weak learners, the computation of feature importance is typically derived from these trees. When a feature is used for splitting in a DT, it provides a certain improvement to the model, often measured by a criterion like the Gini impurity or information gain. The importance of a feature is computed based on the sum of the weighted impurity decrease for all nodes that the feature is used in. In the context of AdaBoost, the feature importance can be computed as a weighted average from all the DTs, where trees that do well on harder-to-classify examples have more weight. This algorithm is implemented by a package in Scikit-learn [38]. We directly used this package and executed it with default parameters.

#### 2.2.2. Categorical Boosting

CatBoost is an open-source, high-performance machine learning library developed by Yandex, a Russian multinational IT company [29]. The name “CatBoost” originates from two key attributes of the algorithm: CATegorical features support and gradient BOOSTing. Unlike traditional gradient boosting techniques, CatBoost uses a variant called “ordered boosting”, which helps prevent overfitting. CatBoost uses a particular type of DT called “oblivious trees”, which are more memory-efficient and faster to evaluate than standard depth-wise trees. CatBoost, as a sophisticated gradient boosting framework, offers tools to compute and visualize feature importance, helping model interpretability and potentially guiding further feature engineering or selection. CatBoost calculates feature importance based on the number of times a feature is used to split the data, weighted by the improvement to the model as a result of those splits. Essentially, the more a feature helps in distinguishing the target variable (thereby reducing the model’s loss), the more important it is considered. We retrieved the program of this algorithm at https://catboost.ai/en/docs/concepts/installation (accessed on 2 March 2023) and executed it with default parameters.

#### 2.2.3. Extremely Randomized Trees

“ExtraTrees” stands for “extremely randomized trees”. It is an ensemble learning method fundamentally similar to RFs but with a key difference in the way splits are made during the construction of the DTs [30]. While RFs only ensure that each bootstrap sample is random, ExtraTrees goes a step further by making the split thresholds random as well. This increased randomness helps in adding diversity to the model. ExtraTrees has an option to either use the whole dataset to grow trees (without bootstrapping) or to use bootstrap samples, similar to RFs. Given that ExtraTrees is based on DTs, the mechanism for determining feature importance is related to the structure of these trees. Every time a split of a node is made on a particular feature, it results in some reduction in impurity (e.g., Gini impurity for classification or mean squared error for regression). The importance of a feature is computed by averaging the reduction in impurity over all the nodes of all the trees where that feature is used for splitting. The program of this algorithm was sourced from Scikit-learn [38]. Default parameters were used.

#### 2.2.4. Least Absolute Shrinkage and Selection Operator

Lasso, which stands for “least absolute shrinkage and selection operator”, is a regression analysis method that performs both variable selection and regularization [31]. In a traditional linear regression, the objective is to minimize the sum of squared residuals. Lasso modifies this objective by adding a penalty for the absolute value of the magnitude of the coefficients, which is called the L1 penalty. Feature importance is closely tied to the coefficients assigned to each feature. The L1 regularization in Lasso tends to force the coefficients of less important features to be exactly zero. This inherent feature selection capability means that any variable with a non-zero coefficient can be considered “selected” or “important” by the Lasso model. In Lasso, the magnitude of a coefficient can serve as an indicator of feature importance. Larger coefficients (either positive or negative) suggest that a feature has a stronger impact on the dependent variable. This makes Lasso a valuable tool, especially in high-dimensional datasets, where feature selection and interpretability are crucial. Here, we used the Lasso program in Scikit-learn [38]. For convenience, it was performed using default parameters.

#### 2.2.5. Light Gradient Boosting Machine

LightGBM, which stands for “Light Gradient Boosting Machine”, is a gradient boosting framework specifically designed to be efficient and scalable [32]. It was developed by Microsoft and has become increasingly popular in the machine learning community for its speed and efficiency, especially in cases with large datasets or when dealing with high-dimensional data. Unlike other boosting algorithms that grow trees level-wise, LightGBM grows trees leaf-wise, meaning it chooses the leaf with the maximum delta loss to grow at each iteration. LightGBM divides continuous feature values into discrete bins, creating histograms which accelerates the training process and reduces memory usage. LightGBM, like other gradient boosting frameworks, offers built-in tools to compute and visualize feature importance. Total gain represents the improvement in accuracy brought by a feature to the model. For every feature, the improvement in the model’s accuracy, or the gain, is accumulated each time that feature is used to split the data in the trees. The total gain brought by a feature can be thought of as the reduction in the loss as a result of adding that feature. Thus, total gain can be used as an indication of feature importance in LightGBM. This study used the LightGBM program reported at https://lightgbm.readthedocs.io/en/latest/ (accessed on 10 May 2020), which was executed with its default parameters.

#### 2.2.6. Monte Carlo Feature Selection

Monte Carlo feature selection (MCFS) is a method proposed by Michal Dramiński, et al. to evaluate the importance of features in datasets [33]. MCFS employs a randomized approach, hence the reference to “Monte Carlo”, a famous methodology for obtaining numerical results through random sampling. By utilizing a Monte Carlo approach and aggregating results across multiple iterations, MCFS can capture complex interactions between features, making it effective for datasets where feature interactions play a crucial role. Here is how MCFS works: (1) Randomization: It begins by constructing multiple subsets of samples randomly. It first selects some feature subsets randomly, and for each feature subset, multiple subsets of samples are constructed randomly. (2) DT construction: For each subset of data, a DT is constructed, much like the process in RF. (3) Scoring: After growing many DTs, the importance of a feature is determined based on how often it is used to split data, the coverage and information gain of those decision nodes involving the feature, and the weighted accuracy of the DTs. The MCFS program was downloaded from http://www.ipipan.eu/staff/m.draminski/mcfs.html (accessed on 4 June 2019), which was also performed by default parameters.

#### 2.2.7. Random Forest

Due to the ensemble nature, RFs can be used to gauge the importance of each feature, which can be critical for understanding, interpreting, and improving machine learning models [34]. When a tree in a RF makes a decision, it uses one feature from the set of available features. Over the course of many trees, some features will be used more often and at earlier decision points, indicating that they are crucial for making accurate predictions. Two common methods to calculate feature importance in RFs are described as follows: (1) Gini importance: For each feature, the RF algorithm computes the average impurity decrease (commonly using the Gini impurity) that results from splits over that feature, normalized by the number of samples that reach the split. The decrease in impurity (or increase in purity) acts as a proxy for the feature’s importance. (2) Permutation importance: After the RF model has been trained, the values of each feature are randomly permuted in the validation set, and the decrease in the model’s accuracy is computed. Features that are more important to the model will produce a larger drop in accuracy when their values are permuted. This algorithm was implemented by a package in Scikit-learn [38], which was directly employed in this study. Likewise, it was performed by default parameters.

#### 2.2.8. Extreme Gradient Boosting

XGBoost, which stands for “extreme gradient boosting”, is an open-source software library that provides a gradient boosting framework [35]. Unlike other gradient boosting algorithms that grow trees by depth (depth-first), XGBoost grows trees by level (level-wise) and then prunes them using depth-first method. This results in a more balanced tree that can generalize better. XGBoost incorporates L1 (Lasso) and L2 (Ridge) regularization terms on the weights, which can prevent overfitting. Given that XGBoost is a gradient boosting framework that primarily uses DTs, it offers several methods by which to determine feature importance: (1) Weight (or frequency) importance: It measures the number of times a feature appears in all the trees in the model, and a feature that appears more frequently is considered to be more important. (2) Gain importance: For each feature, it calculates the improvement in accuracy brought by a feature when it is used in trees. The gain of each feature is averaged over all trees to compute its importance. (3) Coverage importance: This method calculates the relative quantity of observations concerning a feature. Features that affect a large number of observations have higher coverage importance. The XGBoost program used in current study was obtained from https://xgboost.readthedocs.io/en/stable/ (accessed on 2 March 2023). It was also executed using default parameters.

### 2.3. Incremental Feature Selection

The IFS is a feature selection technique that aims to determine the most relevant subset of features by incrementally adding features based on a specified criterion, typically their importance or impact on a model’s performance [36]. Here is how IFS works: (1) rank all features based on a specified criterion; this could be based on univariate statistics, correlation with the target, or model-derived importance scores; (2) start with the most important feature and build a model, evaluating its performance with one cross-validation method [39], yielding a specified metric; (3) add the next-most-important feature then re-build the model with the accumulated feature set, recording the performance; (4) continue the process until all features are added or until a certain stopping criterion is met; this stopping criterion can be a threshold of performance improvement or it can be when performance starts to deteriorate or saturate; (5) determine the feature subset that gives the best performance.

The approach is conceptually simple and can be implemented with minimal effort. It is model-agnostic and can be applied with any machine learning algorithm. However, like other greedy algorithms, it might not always find the global optimum because it relies on local decisions at each step.

### 2.4. Synthetic Minority Oversampling Technique

SMOTE, which stands for “synthetic minority over-sampling technique”, is a popular method used to handle class imbalance in datasets, especially in the context of classification problems [40]. In many real-world scenarios, datasets might have an unequal distribution of classes. Training a model on such imbalanced datasets can lead to poor performance for the minority class because the model tends to become biased towards the majority class. One common way to address this imbalance is by generating synthetic samples in the dataset, and SMOTE is designed specifically for this purpose. Here is how SMOTE works: (1) start by choosing a random sample from the minority class; (2) compute its *k* nearest neighbors (from the minority class) (the number *k* can be a user-defined parameter); (3) randomly select one of the *k* neighbors, compute the difference between the feature vector of the sample under consideration and its chosen neighbor, multiply this difference by a random number between 0 and 1, and then add it to the feature vector of the sample; this creates a new, synthetic data point; (4) repeat steps 1–3 until the desired number of synthetic samples are generated. The present study adopted the SMOTE program available at https://github.com/scikit-learn-contrib/imbalanced-learn (accessed on 24 March 2020). Default parameters were used to execute this program.

### 2.5. Classification Algorithm for Establishing Classification Rules

In our study, two supervised classification algorithms, DT and RF, were chosen to implement the IFS method. These two algorithms have wide applications in tackling different problems in health care [41,42]. The DT algorithm, detailed in [37], uses a hierarchical decision structure for classification, while RF, described in [34], employs multiple DTs to enhance accuracy. Both algorithms aim to optimize the IFS method by refining and selecting significant features to improve model performance.

#### 2.5.1. Decision Tree

A DT is a versatile machine learning algorithm. It is a tree-like structure where each node represents a feature (or attribute), each branch represents a decision rule, and each leaf represents an outcome [37]. The root node represents the entire dataset, which is further divided into two or more homogeneous sets. A decision node represents the decision-making point, where the data are further divided. A leaf node represents the end result or the target variable. Here is how a DT is built: (1) start with the entire dataset at the root; (2) select the best attribute to split the data using an attribute selection measure (ASM) (the commonly used ASMs for this purpose include information gain, Gini index, gain ratio, etc.); (3) make a decision node based on the best attribute; (4) split the dataset into subsets; i.e., split the decision node into sub-nodes; (5) recursively apply steps 2–4 to each child node until one of the stopping criteria is met; e.g., the subset at a node has all the same target values (pure node), the information gain or decrease in impurity is below a threshold, and the number of samples in a node is below a threshold; (6) pruning is used to reduce overfitting, which involves removing parts of the tree that do not provide significant power in predicting target values.

#### 2.5.2. Random Forest

The RF is a versatile and widely used ensemble machine learning algorithm. An ensemble method refers to a technique that combines the predictions from multiple models in order to produce a more robust and accurate result [34]. RF, in particular, builds multiple DTs and merges them together to obtain a more stable and accurate prediction. Here is how RF works: (1) bootstrap samples (with replacement) are drawn from the dataset, and a DT is built based on these samples; (2) for each split in the tree-building process, we only consider a random subset of features, which add another layer of randomness into the RF; (3) steps 1 and 2 are repeated several times; thus, multiple DTs are built; (4) for classification, we take the majority vote from all trees as the prediction result (i.e., the class that obtains the most “votes” from all trees is the final prediction).

The RF often delivers accurate predictions because it is less prone to overfitting than individual DTs. However, while individual DTs are interpretable, a whole forest can be hard to understand and visualize.

In this study, we directly adopted the DT and RF packages in Scikit-learn [38]. These packages were executed using default parameters.

### 2.6. Performance Evaluation

The weighted F1 score is a valuable metric used to assess a classifier’s performance, especially in datasets with class imbalances. Unlike the macro F1 score, which averages the F1 scores [43,44,45,46,47,48,49,50,51,52] of each class, the weighted version allocates weights proportionate to each class’s sample size. This ensures that larger classes have a more pronounced influence on the overall score. By doing so, the weighted F1 score provides a more accurate and nuanced understanding of a classifier’s efficacy across diverse class sizes, highlighting its strengths and weaknesses in varied real-world scenarios. Here is the specific formula for this metric:

(1) $${Precision}_{i}=\frac{{TP}_{i}}{{TP}_{i}+{FP}_{i}},$$

(2) $${Precision}_{weighted}=\sum_{i=1}^{L}{Precision}_{i}\times w_{i},$$

(3) $${Recall}_{i}=\frac{{TP}_{i}}{{TP}_{i}+{FN}_{i}},$$

(4) $${Recall}_{weighted}=\sum_{i=1}^{L}{Recall}_{i}\times w_{i},$$

(5) $$Weighted\;F1=\frac{2\cdot {Precision}_{weighted}\cdot {Recall}_{weighted}}{{Precision}_{weighted}+{Recall}_{weighted}}.$$

In this formula, i denotes each individual class, with $w_{i}$ symbolizing the proportion of samples in that class relative to the overall sample count. L indicates the total number of classes. Additionally, *TP* is an abbreviation for true positives, *FP* means false positives, and *FN* designates false negatives.

Alongside weighted F1 and macro F1 scores, the classification accuracy (ACC) and Matthews correlation coefficient (MCC) [53,54] were also employed to fully display the performance of all models. ACC is defined as the proportion of correctly predicted samples among all samples. MCC is more complex than ACC. Two matrices are constructed in advance, say *X* and *Y*, where *X* stores the true class of each sample and *Y* collects the predicted class of each sample. Then, MCC can be computed by

(6) $$MCC=\frac{cov(X,Y)}{\sqrt{cov(X,X)\cdot cov(Y,Y)}},$$

where cov(X,Y) represents the correlation coefficient of *X* and *Y*.

### 2.7. Outline of the Analysis Procedure

**Figure 1:**
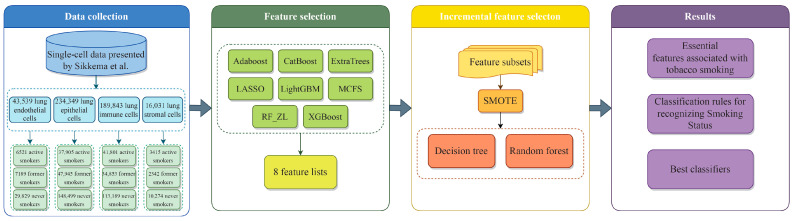
Flow chart of the entire analysis process. We analyzed gene expression profiling data from 43,539 lung endothelial cells, 234,349 lung epithelial cells, 189,843 lung immune cells, and 16,031 lung stromal cells, which included three smoking states: active smoker, former smoker, and never smoker. Using eight feature ranking algorithms, we generated ordered feature sets based on their significance to smoking and integrated them into the IFS framework to yield essential genes, classification rules, and classifiers.

In this study, several machine learning algorithms, described in Section 2.2, Section 2.3, Section 2.4 and Section 2.5, were integrated to analyze the gene expression profiling data of four cell types with three smoking statuses. The data on each cell type were analyzed in the same pipeline. First, the data were analyzed by eight feature ranking algorithms, yielding eight feature lists. Then, each list was fed into the IFS method to extract essential genes, classification rules, and construct best classifiers. At this stage, several feature subsets were generated according to the given feature list. On each feature subset, the data were processed by via SMOTE to tackle the imbalanced problem, and then DT and RF were used to build classifiers on the balanced data. The entire procedure is illustrated in Figure 1.

## 3. Results

In this study, we thoroughly analyzed the effects of smoking status on lung cells, as shown in Figure 1. The methodology involved three primary stages: data collection, feature selection, and the development of classification rules. We sourced single-cell data from CELLxGENE, as documented by Sikkema et al. Upon data collection, we applied eight feature ranking algorithms, each assessing the importance of features. A critical element of our analysis was the implementation of the IFS method, which proved instrumental in pinpointing crucial biomarkers for differentiating smoking status. The culmination of this process was the establishment of quantitative classification rules, which were formulated by synthesizing insights obtained from the IFS with DT. These rules are characterized by their correlation with specific gene expressions, highlighting the significance of these genes in identifying various smoking statuses. The following section details the results obtained at each stage of the study.

### 3.1. Feature Ranking Results of Features in Order of Importance

In this study, we utilized eight advanced feature-ranking algorithms—namely, Adaboost, CatBoost, ExtraTrees, LASSO, LightGBM, MCFS, RF_ZL, and XGBoost—to identify critical gene markers associated with smoking. Each algorithm processed 28,024 gene features, derived from a total of 483,762 distinct lung cells. These included 43,529 endothelial cells, 234,349 epithelial cells, 189,843 immune cells, and 16,031 stromal cells. The features were ranked based on their importance. A comprehensive list of these features, compiled from all algorithms, is presented in Table S2. For convenience, these lists were called Adaboost, CatBoost, ExtraTrees, LASSO, LightGBM, MCFS, RF_ZL, and XGBoost feature lists.

### 3.2. IFS Results and Feature Intersections for Finding Key Features Associated with Smoking Status

**Figure 2:**
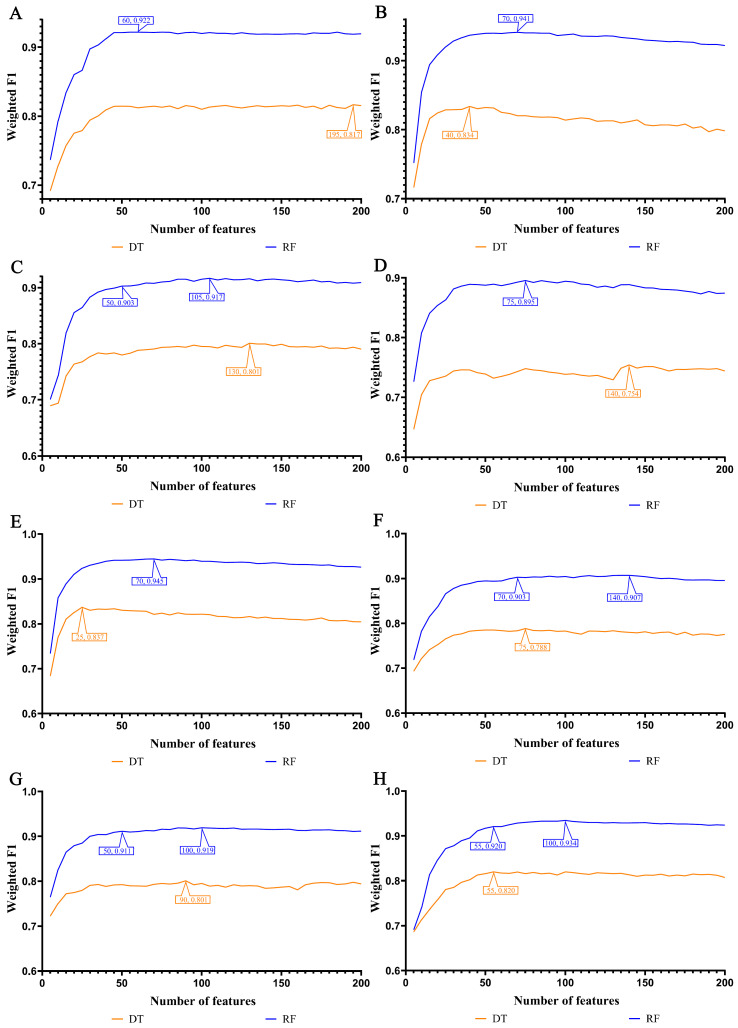
IFS curves for evaluating the performance of the two classification algorithms based on the weighted F1 score in endothelial cells: (**A**) IFS curves based on AdaBoost feature list; (**B**) IFS curves based on CatBoost feature list; (**C**) IFS curves based on ExtraTrees feature list; (**D**) IFS curves based on Lasso feature list; (**E**) IFS curves based on LightGBM feature list; (**F**) IFS curves based on MCFS feature list; (**G**) IFS curves based on RF_ZL feature list; (**H**) IFS curves based on XGBoost feature list.

**Figure 3:**
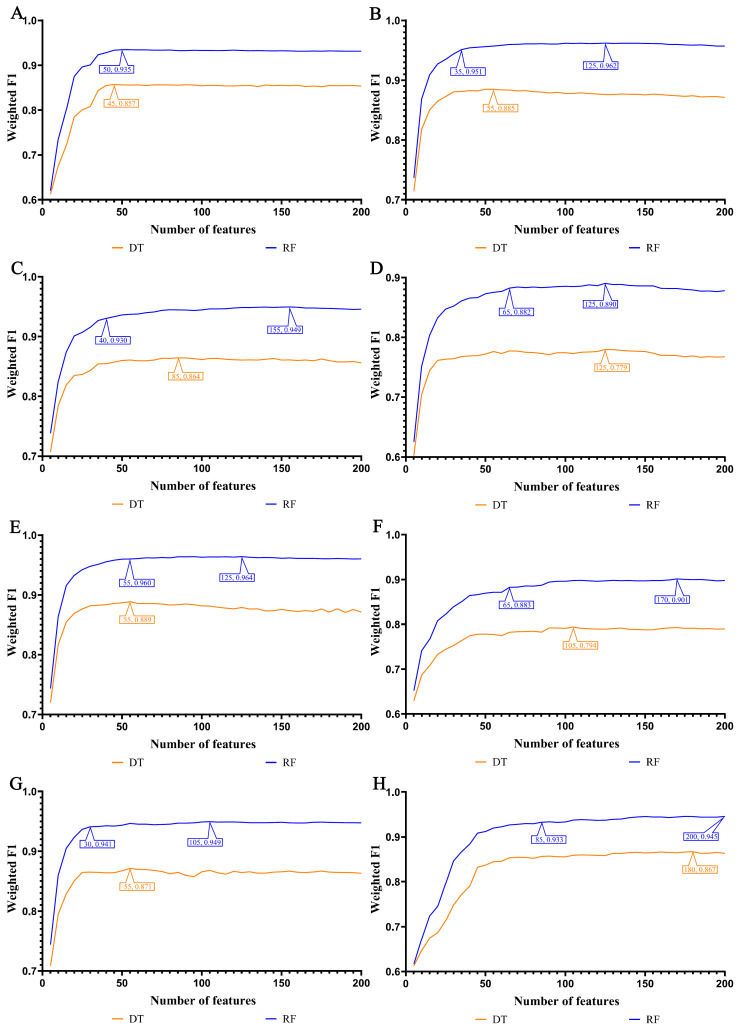
IFS curves for evaluating the performance of the two classification algorithms based on the weighted F1 score in epithelial cells. (**A**) IFS curves based on AdaBoost feature list; (**B**) IFS curves based on CatBoost feature list; (**C**) IFS curves based on ExtraTrees feature list; (**D**) IFS curves based on Lasso feature list; (**E**) IFS curves based on LightGBM feature list; (**F**) IFS curves based on MCFS feature list; (**G**) IFS curves based on RF_ZL feature list; (**H**) IFS curves based on XGBoost feature list.

**Figure 4:**
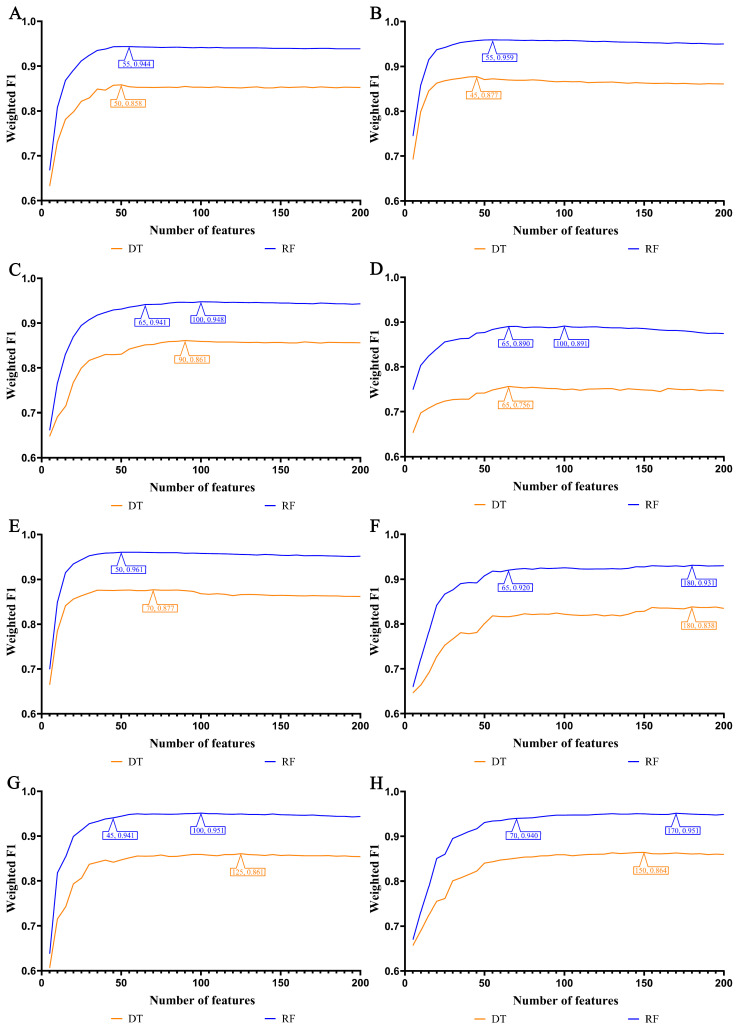
IFS curves for evaluating the performance of the two classification algorithms based on the weighted F1 score in immune cells: (**A**) IFS curves based on AdaBoost feature list; (**B**) IFS curves based on CatBoost feature list; (**C**) IFS curves based on ExtraTrees feature list; (**D**) IFS curves based on Lasso feature list; (**E**) IFS curves based on LightGBM feature list; (**F**) IFS curves based on MCFS feature list; (**G**) IFS curves based on RF_ZL feature list; (**H**) IFS curves based on XGBoost feature list.

**Figure 5:**
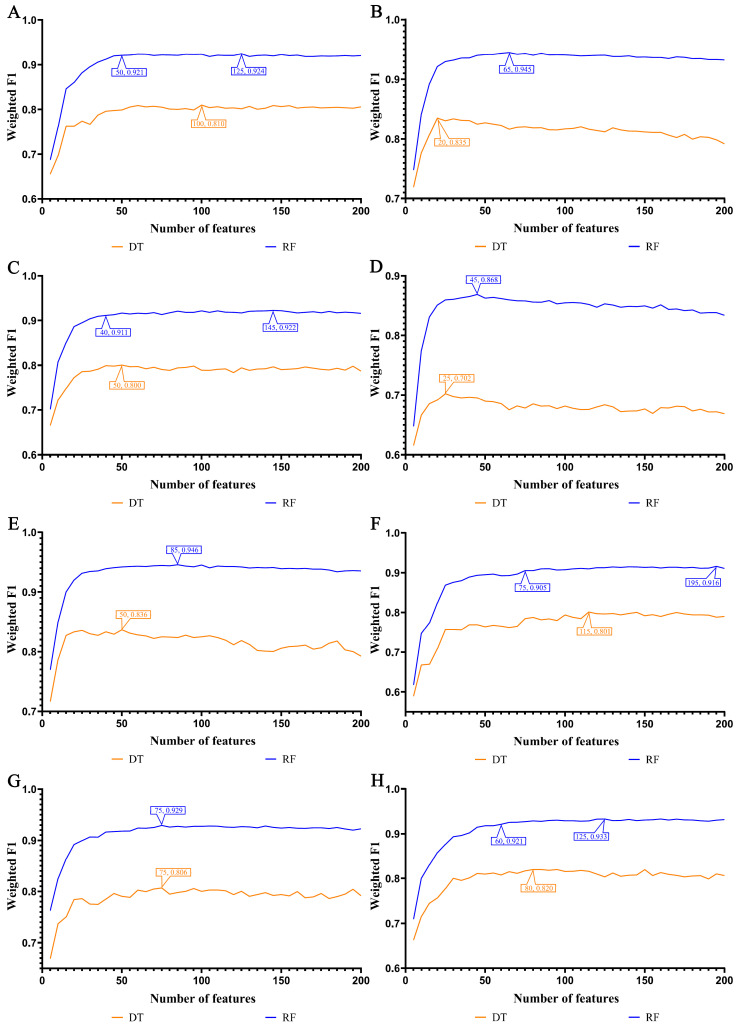
IFS curves for evaluating the performance of the two classification algorithms based on the weighted F1 score in stroma cells: (**A**) IFS curves based on AdaBoost feature list; (**B**) IFS curves based on CatBoost feature list; (**C**) IFS curves based on ExtraTrees feature list; (**D**) IFS curves based on Lasso feature list; (**E**) IFS curves based on LightGBM feature list; (**F**) IFS curves based on MCFS feature list; (**G**) IFS curves based on RF_ZL feature list; (**H**) IFS curves based on XGBoost feature list.

**Figure 6:**
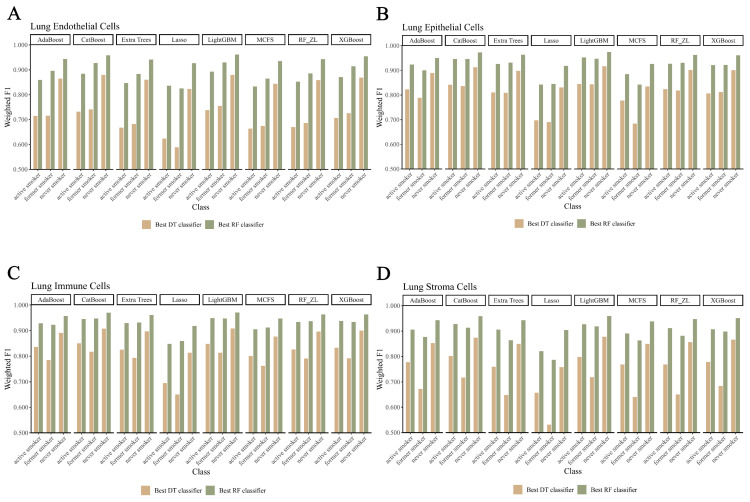
Performance of the optimal classifier built on eight feature lists in three classes. A grouped bar chart is utilized to compare the performance of two classifiers (RF, DT) between three classes for each feature set, using the weighted F1 score as the criterion: (**A**) grouped bar chart based on endothelial cells data; (**B**) grouped bar chart based on epithelial cells data; (**C**) grouped bar chart based on immune cells data; (**D**) grouped bar chart based on stroma cells data.

In our study, we focused on the top 200 features as identified by each of the eight feature ranking algorithms: Adaboost, CatBoost, ExtraTrees, LASSO, LightGBM, MCFS, RF_ZL, and XGBoost. The IFS method, with a step size of 5, was applied to derive feature subsets. The model based on each feature subset was assessed via ten-fold cross-validation. For a comprehensive classification, we used the weighted F1 score to compare the performance of the RF and DT classifiers. The collective evaluation results of these classifiers are presented in Table S3, while Figure 2, Figure 3, Figure 4 and Figure 5 visually depict the IFS outcomes. Additionally, Figure 6 illustrates the performance comparisons between the three classes.

When assessed using the weighted F1 score, machine learning algorithms exhibited resilience across varying feature complexities. The best-performing classifiers for lung endothelial cells were as follows: LightGBM-RF with 70 features (weighted F1: 0.945; Figure 2A) and similarly for other algorithms and cell types, as detailed. The results consistently showed RF’s superior performance over DT across all eight feature sets. Furthermore, Figure 6 compares the performance of classifiers for three classes (active smoker, former smoker, and never smoker), underscoring RF’s dominance over DT. RF’s uniformly high performance across different feature complexities sets a benchmark for future research. 

**Figure 7:**
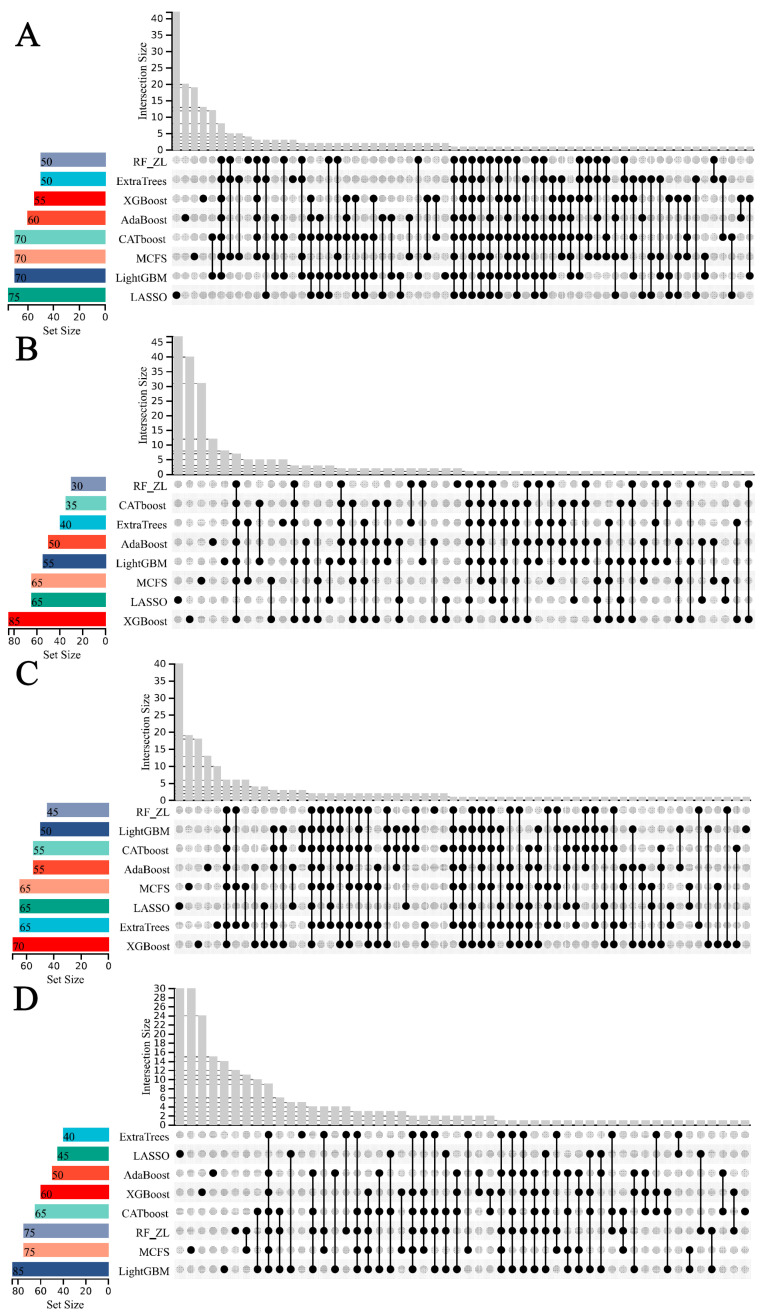
UpSet diagram of the essential feature subsets obtained using eight feature ranking algorithms. “Set Size” is the count of the number of features in each set, “Intersection Size” is the count of the number of features after taking the intersection of the eight feature sets, the black dots indicate the unique features of a feature set, and the line between the dots and the dots indicates the unique intersection of different feature sets. (**A**) UpSet diagram on endothelial cells data. (**B**) UpSet diagram on epithelial cells data. (**C**) UpSet diagram on immune cells data. (**D**) UpSet diagram on stroma cells data.

**Figure 8:**
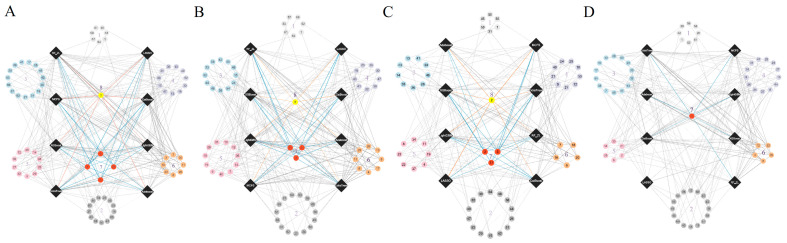
Network Venn diagram of gene feature sets in lung cells to display more detailed intersection. Network Venn diagram illustrates the intersection of optimal gene subsets for lung cells. Black diamond nodes: These nodes represent the optimal gene subsets as identified by eight distinct feature ranking algorithms. Node numbering: Genes that are commonly selected across similar optimal subsets are aggregated into clusters, each designated by a numerical identifier. Node color-coding: Each color signifies the number of optimal subsets that have selected the respective gene set. The color scheme is as follows: light gray for gene sets chosen by one subset; dark gray for two; blue for three; purple for four; pink for five; orange for six; red for seven; yellow for eight subsets. Interconnections (connecting lines): These lines depict the interrelationship between the optimal subsets and their influence in the selection of gene features. Supplementary Information: For detailed insights, including the specific genes amalgamated within each labeled gene set and the exact optimal subset responsible for their selection, see Table S5. (**A**) Network Venn diagram based on endothelial cells data. (**B**) Network Venn diagram based on epithelial cells data. (**C**) Network Venn diagram based on immune cells data. (**D**) Network Venn diagram based on stroma cells data.

In our study, the weighted F1 score was utilized as the primary metric for evaluating classification performance. Pertinent feature subsets, identified by algorithms such as Adaboost, CatBoost, ExtraTrees, LASSO, LightGBM, MCFS, RF_ZL, and XGBoost, were located at the maximum points. These subsets comprised 60, 70, 105, 75, 70, 140, 100, and 100 features for lung endothelial cells; 50, 125, 155, 125, 125, 170, 105, and 200 features for lung epithelial cells; 55, 55, 100, 100, 50, 180, 100, and 170 features for lung immune cells; and 125, 65, 145, 45, 85, 195, 75, and 125 features for lung stroma cells, respectively. It can be found that several maximum points need no less than 100 features. For these points, a relative high point was extracted, which need less features, whereas the weighted F1 was a little lower than the maximum weighted F1. For example, the IFS results with RF on the ExtraTrees feature list indicated that RF reached the maximum weighted F1 (0.917) when top 105 features were used (Figure 2C). The relatively high weighted F1 (0.903) was accessed by using top 50 features, which was also marked in Figure 2C. All such relative high points were marked in the corresponding IFS curves (Figure 2, Figure 3, Figure 4 and Figure 5). The corresponding feature subsets were called essential feature subsets. We conducted an extensive analysis of the interrelations and overlaps among these subsets, visualizing the intersections in upset diagrams (lung endothelial cells, Figure 7A; lung epithelial cells, Figure 7B; lung immune cells, Figure 7C; lung stroma cells, Figure 7D), with detailed data presented in Table S4. Additionally, we created a network Venn diagram by converting features from ENSEMBL ID to gene symbols then intersecting the optimal subsets (lung endothelial cells, Figure 8A; lung epithelial cells, Figure 8B; lung immune cells, Figure 8C; lung stroma cells, Figure 8D), detailed in Table S5. Black diamond nodes represent the optimal subsets from the eight feature ranking algorithms. The numbers on these nodes indicate the gene feature sets selected by the corresponding subsets, with the same color denoting sets chosen by an equal number of optimal subsets. These numbers also indicate the frequency of selection. The connecting lines between nodes illustrate the specific optimal subsets involved in selection. Notably, significant features recurring in multiple subsets were highlighted, emphasizing their crucial role in classifying smoking status.

The identification of key gene features essential for classifying smoking status is extensively discussed in this study. In particular, the discussion section serves as a valuable resource for exploring these biological aspects and their impact on lung cells. It provides a detailed analysis, insights into recent research, and suggests potential directions for future investigations into the harmful effects of tobacco smoking.

### 3.3. Establishing Classification Rules for Identifying Smoking Status

**Figure 9:**
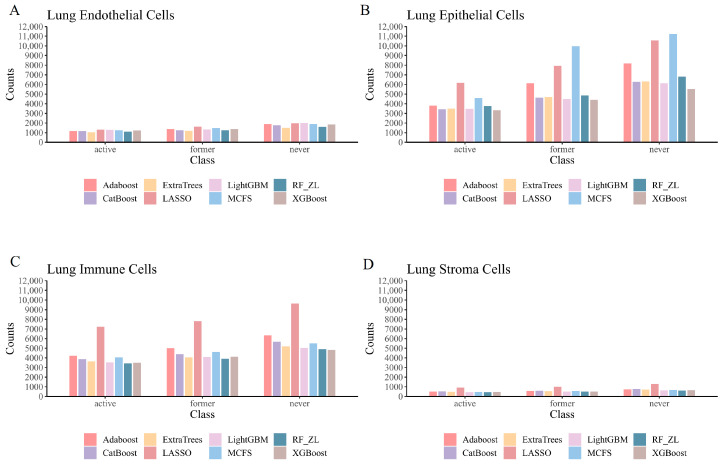
Lollipop plot showing the number of rules for identifying smoking status. In the three predictive classes, the number of rules obtained via DT based on eight different feature subsets is shown. The different colors represent the different feature lists obtained by the eight feature ranking algorithms. (**A**) Lollipop plot on endothelial cells data. (**B**) Lollipop plot on epithelial cells data. (**C**) Lollipop plot on immune cells data. (**D**) Lollipop plot on stroma cells data.

In our study, a DT model, known for its transparency as a white-box machine learning approach, was utilized to develop classification rules. These rules, derived from various measurements, play a crucial role in differentiating smoking statuses, including active smoker, former smoker, and never smoker, as elaborated in Tables S6–S9. Figure 9 demonstrates the rule generation across subsets created by different feature ranking algorithms in four lung cell types. Notably, the total number of rules showed a positive correlation with dataset size, with lung epithelial and immune cells yielding more rules. The most rules were generated from the subset of optimal features identified by LASSO. Furthermore, the rules for identifying “never smokers” were the most numerous, while those for “active smokers” were the fewest. An in-depth analysis of key genetic rules, offering detailed insights into the impact of smoking on lung cells, is presented in Section 4.2.

## 4. Discussion

### 4.1. Analysis of Key Features Associated with Smoking Status

In this study, an exhaustive analysis of single-cell data from four distinct lung cell types was conducted using eight different feature-ranking algorithms. This comprehensive methodology enabled the identification of key genetic features that shed light on the impact of smoking on these cells. These pivotal features, outlined in Table 1, are subject to detailed analysis in this section, and their relevance to the adverse effects of smoking on the lungs is corroborated by current studies. This significant finding enhances our understanding of the mechanisms by which smoking harms the lungs and paves the way for future experimental research into the consequences of smoking.

#### 4.1.1. Qualitative Features in Lung Endothelial Cells

The first identified gene significantly associated with smoking is *B2M* (ENSG00000166710). β-2-Microglobulin (*B2M*) is a minor component of the major histocompatibility class I molecule, present on the membranes of all nucleated cells [55]. The expression levels of *B2M* are influenced by various factors such as gender, age, alcohol consumption, and smoking [56,57]. Notably, serum *B2M* concentrations have exhibited a strong correlation with smoking status; they are highest in never smokers, followed by former smokers, and lowest in current smokers [58]. The underlying mechanism driving this differential expression remains to be elucidated. The above evidence proves the validity of *B2M* as a key feature.

The other two genes, *EEF1A1* (ENSG00000156508) and *TPT1* (ENSG00000133112), are not directly linked to smoking. However, both are associated with non-small-cell lung cancer, exhibiting downregulation in this condition [59]. Smoking is a predominant risk factor for non-small-cell lung cancer [60]. The *EEF1A1* gene encodes an isoform of the alpha subunit in the elongation factor-1 complex, pivotal in transporting aminoacyl tRNAs to the ribosome [61]. Moreover, *EEF1A1* has been associated with lung cancer development in smokers [62]. The *TPT1* gene produces the cytoplasmic protein TCTP, a robust anti-apoptotic factor linked with malignant cell transformation [63]. *TPT1* has also been detected in endothelial cells undergoing apoptosis [64], suggesting that *TPT1* dysregulation in endothelial cells post-smoking may influence apoptosis. Further research is needed to elucidate the precise mechanisms and effects of these genes’ dysregulation in lung endothelial cells following tobacco exposure.

#### 4.1.2. Qualitative Features in Lung Epithelial Cells

The first key feature is the *FTL* gene (ENSG00000087086), which encodes for the light subunit of ferritin. Elevated *FTL* expression levels are linked to irritations caused by cigarette smoke and the onset of chronic obstructive pulmonary disease (COPD). Notably, smoking stands as a primary controllable risk factor for COPD [65]. Studies indicate a pronounced effect of cigarette smoke on small airway epithelial cell populations, evidenced by notable shifts in expression levels contingent on smoking status. Specifically, there is a marked increase in *FTL* expression when stimulated with cigarette smoke extracts [26,66], whereas expression diminishes post smoking cessation [67]. However, the precise mechanism underlying these differential expression patterns is yet to be determined. Collectively, changes in *FTL* expression might serve as an indicator of an individual’s smoking status.

The *MT-ATP8* gene (ENSG00000228253) emerged as another significant gene related to smoking status. Encoded by *MT-ATP8* is a protein integral to ATP synthase. This enzyme, commonly referred to as complex V, constitutes a vital component of the mitochondrial respiratory chain and is instrumental in catalyzing the transformation of ADP into ATP [68]. It is noteworthy that approximately 20% of smokers are predisposed to develop COPD, and data suggest an upregulation of the *MT-ATP8* gene in COPD patients [69]. Moreover, exposure to diesel waste correlates with reduced methylation levels of *MT-ATP8*, with elemental carbon, organic carbon, and PM2.5 also influencing this effect [70]. Given that cigarette smoke is a primary source of PM2.5, organic carbon, elemental carbon, and various detrimental compounds, an association between *MT-ATP8* expression and smoking status is insinuated. Yet, a direct relationship between *MT-ATP8* and smoking necessitates further empirical validation. 

#### 4.1.3. Qualitative Features in Lung Immune Cells

In lung immune cells, we extensively analyze two pivotal genes, *HLA-B* (ENSG00000234745) and *HLA-C* (ENSG00000204525), in relation to smoking status. Both *HLA-B* and *HLA-C* are integral to the human leukocyte antigen (HLA) system, a sophisticated gene set situated on human chromosome 6. This system plays an indispensable role in immune response, primarily facilitating antigen presentation to T cells [71]. Within the scope of the HLA system, multiple studies have elucidated intricate interactions between certain HLA alleles, like HLA-DR, and cigarette smoking, especially in autoimmune disorders akin to rheumatoid arthritis [72,73]. Notably, *HLA-B* and *HLA-C* are linked to smoking-associated ailments, including COPD and lung cancer [74,75]. A particular study revealed that MHC-I molecules, encompassing *HLA-B* and *HLA-C*, exhibited reduced expression in lung adenocarcinoma patients who were non-smokers compared to those who smoked [76]. However, exhaustive investigations are still required to unravel the explicit mechanisms governing the influence of smoking on *HLA-B* and *HLA-C* expression. 

#### 4.1.4. Qualitative Features in Lung Stroma Cells

*HSP90B1* (ENSG00000166598) emerged as the first smoking-associated feature in lung stromal cells. The *HSP90B1* gene is vital for regulating protein folding within the endoplasmic reticulum [77], particularly under stressful conditions. Given that tobacco smoke is replete with reactive oxygen species (ROS) that can induce cellular oxidative stress [78], this may influence the dysregulation of *HSP90B1*. Significantly, a heightened expression of *HSP90B1* was observed in non-smoking patients compared to NSCLC patients with a prior smoking history [79]. While experimental validation is essential to ascertain the exact mechanisms by which smoking impacts *HSP90B1*, this gene holds potential as a diagnostic marker for discerning smoking status.

*LCN2* (ENSG00000148346) emerged as another key gene related to lung stromal cells. This gene is responsible for encoding the NGAL protein, essential in regulating innate immunity and iron homeostasis [80]. Research indicates that cigarette smoke significantly alters *LCN2* expression, suggesting its potential as a marker for the harmful effects of smoke exposure [81]. Notably, *LCN2* expression markedly increased in rat and mouse lungs after 13 and 6 months of cigarette smoke exposure, respectively [82,83]. Additionally, *LCN2* is posited as a prospective prognostic biomarker, potentially signaling an increased metastatic disease risk among female smokers [84]. Hence, *LCN2* might serve as an insightful genetic indicator for discerning smoking status.

The discovery of specific genes as indicators of smoking status in lung cells greatly enhances our grasp of how smoking impacts lung health. This breakthrough paves the way for early detection of smoking-induced damage, personalized treatments, and targeted public health strategies based on genetic risk. Additionally, it supports further genetic research into lung diseases and the development of diagnostic tools for precise evaluation of smoking’s molecular effects.

### 4.2. Analysis of Decision Rules for Indicating Smoking Status in Different Lung Cell Types

In this section, we analyze the classification rules set forth for distinguishing between active smokers, former smokers, and never smokers. Depending on the cell type under consideration, we scrutinize several key gene parameters that show strong correlation with differential expression relative to smoking status. Recent scholarly publications support the reliability of these parameters.

#### 4.2.1. Qualitative Rule Parameters in Lung Endothelial Cells

In lung endothelial cells, we detail three important gene parameters, ENSG00000130208 (*APOC1*), ENSG00000166710 (*B2M*), and ENSG00000127528 (*KLF2*), whose expression levels help to identify different smoking statuses.

In rules, *APOC1* expression is notably down-regulated in active smokers and up-regulated in former smokers. *APOC1*, a protein present in lipoproteins, plays a crucial role in lipid metabolism by facilitating the transport of fats in the body [85,86]. Active smoking can perturb the equilibrium of lipoproteins, particularly low-density lipoproteins [87,88], potentially leading to changes in the expression of genes such as *APOC1*. A recent investigation determined that SNP rs4420638, situated downstream of the *APOC1* gene [89], is significantly associated with smoking cessation. This underlines the correlation between *APOC1* expression and smoking status, suggesting the potential utility of *APOC1* in differentiating between smoking states.

In rules established to determine smoking status, the expression level of *B2M* was observed to be lowest in active smokers, intermediate in former smokers, and highest in individuals who have never smoked. The relationship between *B2M* expression levels and smoking status is detailed in Section 4.1.1, with the outcomes of various studies aligning with our findings. Consequently, *B2M* can serve as a pivotal parameter in formulating rules to discern smoking status.

*KLF2* stands out as another pivotal parameter. Its expression is up-regulated in rules that identify active smokers and down-regulated in those distinguishing former smokers. *KLF2*, commonly known as Krüppel-like factor 2, is integral to various processes, including angiogenesis, regulation of blood vessel tone, ifnflammatory responses, and lymphocyte development and migration [90]. Cigarette smoke, given its essential role in endothelial function and its anti-inflammatory and vasoprotective properties [91], impairs endothelial function, affecting the inner lining of blood vessels vital for vascular health [92]. In summary, *KLF2* can be used as an important constituent parameter in rules for recognizing smoking status.

#### 4.2.2. Qualitative Rule Parameters in Lung Epithelial Cells

In lung epithelial cells, we detail two important gene parameters, ENSG00000170345 (*FOS*) and ENSG00000229807 (*XIST*), whose expression levels help to identify different smoking statuses. 

In rules established to determine smoking status, the expression of *FOS*, when present as a parameter, was highest in rules for active smokers, intermediate for former smokers, and lowest for those who have never smoked. *FOS* is a transcription factor that combines with JUN to form the AP-1 complex, modulating gene transcription. It is engaged in a multitude of cellular processes and acts as an immediate early gene, promptly activated in response to various stimuli [93]. Cigarette smoke, laden with noxious chemicals, induces cellular stress and damage. This may prompt cells to invoke pathways, such as the *FOS* gene, either for response and repair mechanisms or for programmed cell death [94]. Furthermore, smoking systemically incites chronic inflammation in the lungs, potentially stimulating pathways that elevate FOS (c-Fos) expression as a cellular countermeasure [95,96]. One particular study underscored that the anomalous lung epithelial–mesenchymal transition and cell proliferation due to tobacco smoke were linked to the up-regulation of phosphorylated p38 and phosphorylated c-Fos, substantiating the relationship between *FOS* expression and smoking [97]. Hence, *FOS* is instrumental as a key parameter in formulating rules to ascertain smoking status.

*XIST* emerges as another significant parameter. Its expression is up-regulated in rules identifying former smokers and down-regulated in those distinguishing never smokers. The *XIST* gene synthesizes a non-coding RNA responsible for silencing one of the two X chromosomes in females, thus ensuring gene expression parity between males and females [98]. Smoking has the potential to modify hormone levels, which, in turn, could influence genes such as *XIST*. The differential *XIST* expression between ex-smokers and never smokers may be associated with these hormonal fluctuations [99,100]. Chen et al. reported that *XIST* enhances apoptosis and inflammatory responses triggered by cigarette smoke extracts via the miR-200c-3p/EGR3 axis [101]. As a result, ex-smokers might exhibit increased *XIST* expression relative to never smokers. Given the established relationship between *XIST* expression and smoking, *XIST* can be rightly considered a vital parameter in the rules to discern smoking status.

#### 4.2.3. Qualitative Rule Parameters in Lung Immune Cells

In lung immune cells, we detail two important gene parameters, ENSG00000100292 (*HMOX1*) and ENSG00000149021 (*SCGB1A1*), whose expression levels help to identify different smoking statuses.

Expression of *HMOX1*, a key parameter used in immune cells to recognize smoking status, was lowest in never smokers and slightly higher in former smokers. Expression of *HMOX1*, a key parameter used in immune cells to recognize smoking status, was lowest in never smokers and slightly higher in former smokers. *HMOX1* (heme oxygenase 1) is an enzyme that degrades heme, producing biliverdin, free iron, and carbon monoxide, and plays a protective role against oxidative stress and inflammation in cells [102,103]. Cigarette smoke contains harmful substances that generate oxidative stress in cells [104], leading to increased expression of *HMOX1* as a protective response [103]. Former smokers may have higher *HMOX1* expression due to their cells adapting to past-smoking-induced oxidative stress. Moreover, smoking can cause long-term lung inflammation [105], and the increased expression of *HMOX1* in smokers or former smokers might be a natural response to reduce this inflammation due to *HMOX1*’s anti-inflammatory effects [106]. For these reasons, *HMOX1* can be used as a key parameter for composing rules that recognize former smokers and non-smokers.

*SCGB1A1* stands out as a crucial rule parameter within immune cells, designed to discern smoking status. Its expression is notably reduced in active smokers compared to slightly elevated levels in former smokers. Also known as CC10 or Uteroglobin, *SCGB1A1* is a protein-coding gene predominantly secreted by Clara cells in the lungs. It boasts anti-inflammatory, immune-modulating, and phospholipase A2 inhibitory functions [107]. Exposure to tobacco smoke can directly harm Clara cells [108], culminating in a diminished production of the *SCGB1A1* protein. The inflammation induced by smoking, especially in the lungs, can further compromise Clara cells, leading to a decline in *SCGB1A1* expression [109]. As an immunomodulator, *SCGB1A1* safeguards the lungs from toxic injuries and preserves respiratory balance [110]. Therefore, *SCGB1A1*’s expression profile offers insights to differentiate active smokers from former smokers. In essence, the varying expression of *SCGB1A1* is a pivotal marker for formulating criteria related to smoking status.

#### 4.2.4. Qualitative Rule Parameters in Lung Stroma Cells

In lung stroma cells, we detail one important gene parameter, ENSG00000166598 (*HSP90B1*), whose expression levels help to identify different smoking statuses.

*HSP90B1* expression, pivotal in lung stromal cells for consistently determining smoking status, is most pronounced in active smokers, then in former smokers, and least in never smokers. As *HSP90B1* is primarily localized in the endoplasmic reticulum (ER), its upregulation may be part of the unfolded protein response, aiding in protein folding and degradation [111]. The variances in *HSP90B1* expression across active, former, and never smokers in lung stromal cells could be a reflection of the body’s adaptive mechanisms to cope with smoking-induced stress, particularly ER stress [112]. Moreover, evidence highlighting the elevated expression of *HSP90B1* in non-smokers diagnosed with non-small-cell lung cancer is detailed in Section 4.1.4. In sum, *HSP90B1* is a crucial marker when formulating criteria to discern smoking status within lung stromal cells.

## 5. Conclusions

In this study, we analyzed gene expression data from four lung cell types using sophisticated machine learning algorithms. This analysis identified a set of genes, including *B2M*, *FTL*, *HLA-B*, and *HSP90B1*, potentially associated with smoking in four types of lung cells. Employing the IFS method within our computational framework, we optimized feature selection for classification. Additionally, we established quantitative rules to determine smoking status, encompassing active smokers, former smokers, and never smokers. Our work not only highlights the precision of machine learning in dissecting lung cell functions but also deepens our understanding of how smoking influences cellular mechanisms at a molecular level. By establishing quantitative rules for smoking status identification, we pave new avenues for targeted research into the cellular consequences of tobacco exposure. Looking forward, this study sets a solid foundation for future inquiries into tobacco’s molecular damage, aiming to unravel the intricate web of its effects on lung health and potential pathways for intervention. Our findings should stimulate a closer examination of the identified biomarkers, promising a more personalized approach in treating and preventing smoking-related lung conditions. However, the findings reported in this study still need solid experiments. They provide valuable clues for experimenters in designing further investigations. 

## Figures and Tables

**Table 1:** Key genes associated with tobacco smoking identified by feature ranking algorithms.

Cell Type	ENSEMBL ID	Gene Symbol	Description
lung endothelial cells	ENSG00000166710	*B2M*	beta-2-microglobulin
	ENSG00000156508	*EEF1A1*	eukaryotic translation elongation factor 1 alpha 1
	ENSG00000133112	*TPT1*	tumor protein, translationally-controlled 1
lung epithelial cells	ENSG00000087086	*FTL*	ferritin light chain
	ENSG00000228253	*MT-ATP8*	mitochondrially encoded ATP synthase 8
lung immune cells	ENSG00000234745	*HLA-B*	major histocompatibility complex, class I, B
	ENSG00000204525	*HLA-C*	major histocompatibility complex, class I, C
lung stroma cells	ENSG00000166598	*HSP90B1*	heat shock protein 90 beta family member 1
	ENSG00000148346	*LCN2*	lipocalin 2

## Data Availability

The data presented in this study are openly available in the CELLxGENE at https://cellxgene.cziscience.com/collections/6f6d381a-7701-4781-935c-db10d30de293 (accessed on 15 August 2023) [27].

## Supplementary Materials

The following supporting information can be downloaded at https://www.mdpi.com/article/10.3390/life14040502/s1: Table S1: Cells included in the specific four cell types. Table S2: Feature ranking results obtained using Adaboost, CatBoost, ExtraTrees, LASSO, LightGBM, MCFS, RF_ZL and XGBoost; Table S3: Performance of IFS with two different classification algorithms on Adaboost, CatBoost, ExtraTrees, LASSO, LightGBM, MCFS, RF_ZL and XGBoost feature lists; Table S4: Intersection of eight gene sets extracted from the Adaboost, CatBoost, ExtraTrees, LASSO, LightGBM, MCFS, RF_ZL and XGBoost feature lists; Table S5: Detailed enumeration of gene features and optimal subset selections, correlating specific genes with their respective selecting subsets from the Adaboost, CatBoost, ExtraTrees, LASSO, LightGBM, MCFS, RF_ZL, and XGBoost optimal feature lists; Table S6: Classification rules generated by decision tree using its optimal features on eight feature lists based on lung endothelial cells; Table S7: Classification rules generated by decision tree using its optimal features on eight feature lists based on lung epithelial cells; Table S8: Classification rules generated by decision tree using its optimal features on eight feature lists based on lung immune cells; Table S9: Classification rules generated by decision tree using its optimal features on eight feature lists based on lung stroma cells.
